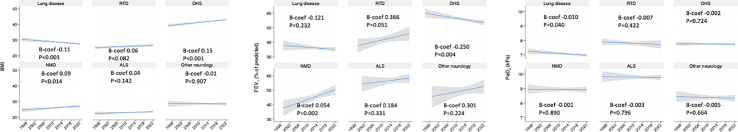# Evolution of Home Mechanical Ventilation in Sweden Over 27 Years: Changing Trends in Incidence, Prevalence, and Patient Characteristics - The Population-Based the Course of Disease in Patients Reported to the Swedish CPAP Oxygen and Ventilator Registry (DISCOVERY) Study

**DOI:** 10.1016/j.chpulm.2024.100108

**Published:** 2024-09-21

**Authors:** Andreas Palm, Ludger Grote, Jonas Einarsson, Daniel Hansson, Mirjam Ljunggren, Josefin Sundh, Magnus Ekström

**Affiliations:** a Department of Medical Sciences, Respiratory, Allergy and Sleep Research, Uppsala University, Uppsala, Sweden; b Centre for Sleep and Wake Disorders, Sahlgrenska Academy, Gothenburg University, Gothenburg, Sweden; c Pulmonary Department, Sahlgrenska University Hospital, Gothenburg, Sweden; d Department of Clinical Sciences, Respiratory Medicine, Allergology and Palliative Medicine, Faculty of Medicine, Lund University, Lund, Sweden; e Department of Respiratory Medicine, Faculty of Medicine and Health, Örebro University, Örebro, Sweden

**Keywords:** HMV, incidence, patient characteristics, prevalence, time trends

## Abstract

**Background:**

Home mechanical ventilation (HMV), noninvasive ventilation and invasive ventilation outside a hospital setting, is a key treatment to improve outcomes in chronic hypoventilation.

**Research Question:**

What are the temporal trends observed over 27 years in Sweden regarding the incidence, prevalence, diagnostic spectrum, and patient characteristics associated with HMV?

**Study Design and Methods:**

This was a national population-based longitudinal analysis of the Course of Disease in Patients Reported to the Swedish CPAP Oxygen and Ventilator Registry (DISCOVERY) study of patients initiating HMV between 1996 and 2022. Time trends stratified by the underlying diagnosis group (lung disease, predominantly COPD, restrictive thoracal diseases, obesity hypoventilation syndrome [OHS], neuromuscular diseases, amyotrophic lateral sclerosis, and other neurologic disorders) were analyzed using linear regression models.

**Results:**

We included 10,555 patients aged ≥ 16 years (mean age 63 [SD, 15] years; 50% women). Between 1996 and 1998 and 2020 and 2022, the HMV incidence increased threefold to 7 per 100,000 people, and the prevalence increased sixfold to 33 per 100,000 people. The most common indication for incident HMV shifted from restrictive thoracal diseases (35% in 1996-1998 to 3% in 2020-2022) to lung disease (14% to 31%), OHS (23% to 33%), and amyotrophic lateral sclerosis (4% to 14%) by 2020 to 2022 (*P* < .001). The proportion of women increased from 47% to 54% (*P* < .013) and the age at initiation of HMV increased from 58 [SD, 15] to 66 [SD, 14] years (*P* < .001). Lung function measured as vital capacity at treatment start increased significantly in all diagnosis groups except for OHS, where both vital capacity and FEV_1_ decreased. In the registry’s first and last 3-year periods, the proportion of patients ventilated invasively decreased from 10% to 2% (*P* < .001).

**Interpretation:**

In the 27 years until 2022, the incidence and prevalence of HMV in Sweden have increased markedly, patient demographics have changed, and use of invasive ventilation has decreased. The average age of patients initiated on HMV has increased, but treatment is started earlier in the disease trajectory.

**Take-home Points**

**Study Question:** What are the temporal trends observed over 27 years in Sweden regarding the incidence, prevalence, diagnostic spectrum, and patient characteristics associated with HMV?

**Results:** Our results indicate that the incidence of HMV is increasing, and the proportion of patients ventilated invasively is decreasing.

**Interpretation:** This study shows that lung disease and obesity hypoventilation syndrome are now the leading indications for HMV. The average age of patients initiated on HMV has increased, but treatment is started earlier in the disease trajectory.

Home mechanical ventilation (HMV) is defined as noninvasive ventilation and invasive ventilation outside a hospital setting. HMV prolongs survival^1^ and improves quality of life^2^ in patients with chronic hypercapnic respiratory failure. The patient groups receiving HMV are heterogeneous, and the etiology of elevated Paco_2_ levels is usually of extrapulmonary origin. The patient panorama is in continuous change. Restrictive thoracic disease (RTD) (ie, kyphoscoliosis, status post-polio, status post-TB) has historically been the leading cause of HMV. Those diseases have decreasing prevalence numbers.^3^^,^^4^ In contrast, the prevalence of obesity is increasing,^5^ leading to an increasing number of patients receiving HMV for obesity hypoventilation syndrome (OHS) with and without OSA.^6^^,^^7^ However, it has been proposed that people with OHS and a high contribution of OSA but only mildly deteriorated blood gases could preferably be treated with CPAP instead of HMV.^8^ With randomized trials showing treatment benefits,^9^, ^10^, ^11^ HMV in patients with amyotrophic lateral sclerosis (ALS) and COPD has gained support. Subsequently, the prevalence rates of HMV are increasing in these two indications. Thereby, a continuous shift in the diagnosis panorama of patients receiving HMV has been reported.^12^, ^13^, ^14^, ^15^ However, data on the change in patient characteristics and addressing time trends in HMV are sparse.

In Sweden, patients with HMV have been reported to the Swedish National Registry for Respiratory Failure (Swedevox) since 1996,^16^ providing unique possibilities to study HMV treatment longitudinally. During the past decade, approximately 600 new patients aged ≥ 16 years have been registered annually with detailed patient characteristics data, allowing us to analyze the patient group over time.

This study aimed to evaluate time trends over 27 years in the incidence, prevalence, diagnosis spectrum, and key clinical characteristics of patients with HMV in Sweden.

## Studey Design and Methods

### Study Design and Population

This was a national population-based longitudinal study of the Course of Disease in Patients Reported to the Swedish CPAP Oxygen and Ventilator Registry (DISCOVERY) study cohort.^17^ In short, DISCOVERY is based on patients reported to Swedevox, cross-linked with several other national registries. In this study, we analyzed data reported to Swedevox from patients aged ≥ 16 years initiating HMV between January 1, 1996, and December 31, 2022.

### Assessments

According to previously published classifications,^17^ primary and secondary underlying diseases were grouped into lung disease (COPD and other lung diseases), RTD (after polio, idiopathic scoliosis, and TB sequelae), neuromuscular disease (NMD) (spinal muscular dystrophia, Duchenne/Becker dystrophia, other neuropathies/myopathies, and myotonic dystrophia), OHS, ALS, and other neurology (spinal cord injury, brain damage/disease, and central hypoventilation). Due to its bad prognosis, ALS is regarded as a disease separate from other neurologic/neuromuscular conditions. Swedevox also includes data on age, sex, weight, height, spirometry values (vital capacity [VC] and FEV_1_) at HMV initiation, information on daytime sleepiness rated using the Epworth Sleepiness Scale,^18^ and arterial blood gas values (Pao_2_ and Paco_2_) at baseline at a scheduled 1-year follow-up. The study is reported by the Strengthening the Reporting of Observational Studies in Epidemiology guidelines.^19^

### Statistical Analyses

Normally distributed continuous data were expressed as mean [SD]. Categorical data were presented as frequencies and percentages. Baseline data are presented both for the time period 2020 to 2022 and for the entire study period. Time trends over 27 years in age, BMI, blood gas, and spirometry values at the initiation of HMV were analyzed using univariable linear regression models. The diagnosis distribution and baseline patient characteristics of patients on HMV on invasive ventilation throughout the study period were analyzed using χ^2^ tests and Student *t* tests, comparing the time periods 1996 to 1998 and 2020 to 2022. Due to the small number of patients when analyzing invasive ventilation use, in this analysis, the first 5-year period of the study period was compared with the last. *P* < .05 was considered statistically significant. Statistical analyses were conducted using Stata software, version 18.0 (StataCorp LP).

### Ethical Considerations

The study was approved by the ethical board of Lund university (Log No. 2018/51), and by the Swedish Ethical Review Authority (2019/01420, 2020/02721, 2021/04984, 2022/00745, 2022/02012), which waived individual consent for the use of collected registry data.

## Results

**Figure 1:**
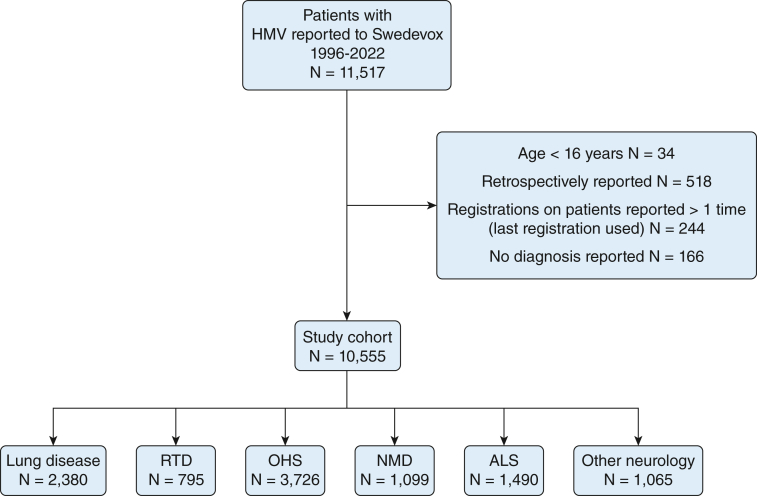
Flowchart of the study population in analyses of time trends in patient characteristics. ALS = amyotrophic lateral sclerosis; HMV = home mechanical ventilation; NMD = neuromuscular disease; OHS = obesity hypoventilation syndrome; RTD = restrictive thoracic disease; Swedevox = Swedish National Registry for Respiratory Failure.

The study cohort consisted of 10,555 prospective patients ≥ 16 years of age (50% women; mean age 63 [SD, 15] years) reported to Swedevox between January 1, 1996, and December 31, 2022 (Fig 1). During the study period, 44 centers reported patients to the registry; of those, 27 reported a total of > 100 patients. Three regions in Sweden, with approximately 600,000 inhabitants, have not reported any patients to the registry in the last 3 years (Västerbotten, Värmland, and Gotland) due to down prioritization of work with registries, and those counties were excluded from incidence and prevalence analyses. Time trend analyses excluded patients aged < 16 years at HMV initiation, those retrospectively reported (n = 515), those reported more than once (n = 244), and those with missing diagnoses (n = 155). Baseline data for the time period 2020 to 2022 and for the entire study period are shown in Table 1 and e-Table 1, respectively. The diagnosis spectrum is shown in Table 2.

**Table 1:** Patient Characteristics at the Initiation of Home Mechanical Ventilation in Sweden Stratified by Main Diagnosis Group, 2020 to 2022

Characteristic	Lung Disease	RTD	OHS	NMD	ALS	Other Neurology	Total
	(n = 577)	(n = 54)	(n = 608)	(n = 177)	(n = 262)	(n = 179)	(n = 1,857)
Women	392 (67.9)	38 (70.4)	320 (52.6)	77 (43.5)	108 (41.2)	69 (38.5)	1,004 (54.1)
Age, y	71.6 [8.7]	69.4 [15.1]	64.0 [13.5]	53.2 [17.2]	66.2 [10.8]	57.8 [18.6]	65.2 [14.2]
BMI, kg/m^2^	27.6 [8.0]	25.9 [5.3]	43.1 [9.2]	26.7 [5.4]	23.7 [4.8]	27.4 [6.9]	32.4 [11.1]
VC, % predicted	51.1 [15.6]	44.6 [21.1]	52.9 [17.0]	45.2 [21.2]	49.8 [18.6]	55.6 [27.9]	50.9 [18.4]
FEV_1_, % predicted	35.2 [17.9]	48.5 [25.3]	52.7 [19.6]	49.6 [22.9]	56.5 [20.2]	56.5 [25.5]	47.1 [21.9]
Pao_2_ at air breathing	7.1 [1.3]	7.7 [1.6]	7.7 [1.4]	8.8 [1.7]	9.7 [1.7]	8.5 [1.9]	7.9 [1.7]
Paco_2_ at air breathing	7.5 [1.1]	7.4 [1.9]	7.0 [1.2]	6.6 [1.2]	6.3 [1.3]	6.8 [1.4]	7.0 [1.3]
Base excess	9.0 [4.6]	7.1 [5.4]	6.9 [4.4]	5.1 [4.3]	5.5 [4.2]	6.0 [4.7]	7.1 [4.7]

Data are presented as No. (%) and mean [SD]. ALS = amyotrophic lateral sclerosis; NMD = neuromuscular disease; OHS = obesity hypoventilation syndrome; RTD = restrictive thoracic disease; VC = vital capacity.

**Table 2:** Distribution of Main Diagnosis Groups and Diagnoses in the Course of Disease in Patients Reported to the Swedish CPAP Oxygen and Ventilator Registry Cohort, 1996 to 2022 (N = 10,555)

Main Diagnosis Group	Diagnosis	No. (%)
Lung disease (n = 2,380)	COPD	1,934 (81)
	Other lung disease	446 (19)
Restrictive thoracal disease (n = 795)	After polio	267 (34)
	Idiopathic scoliosis	365 (46)
	TB sequelae	163 (20)
OHS	OHS with or without OSA	3,726
Neuromuscular disease (n = 1,099)	SMA	63 (6)
	Duchenne/Becker	164 (15)
	Myotonic dystrophy	266 (24)
	Other neuropathy/myop	606 (55)
ALS	ALS	1,490
Other neurologic disease (n = 1,065)	Spinal cord injury	193 (18)
	Brain damage/disease	170 (16)
	Central hypoventilation	81 (8)
	Other diagnosis	621 (58)
Total		10,555

ALS = amyotrophic lateral sclerosis; OHS = obesity hypoventilation syndrome. SMA = spinal muscle atrophia.

### Incidence and Prevalence of HMV in Sweden

**Figure 2:**
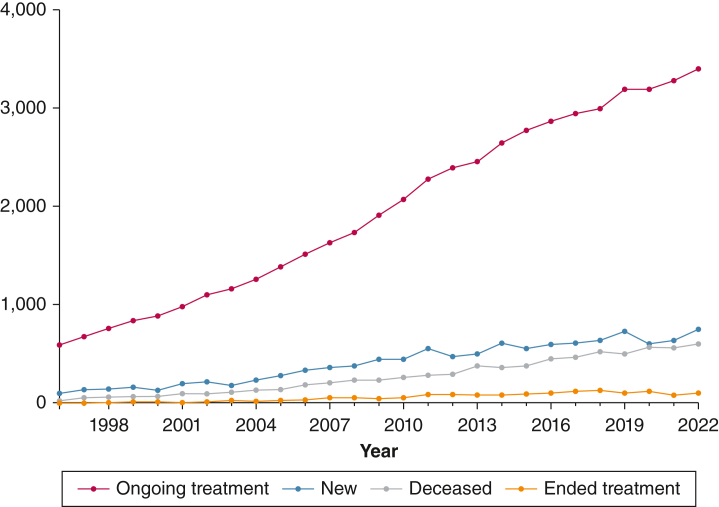
Number of patients with incident and ongoing home mechanical ventilation (HMV) and HMV discontinuation due to mortality and other reasons in Sweden, 1996 to 2022.

Sweden has 10.5 million inhabitants, and the mean number of reported annual HMV initiations in 2020 to 2022 was 648. The mean incidence of HMV therapy increased from 1.4 (1996-1998) to 7 per 100,000 inhabitants (2020-2022). Corresponding prevalence numbers for patients with ongoing HMV therapy increased from 517 (December 1995) to 3,390 by December 2022 (Fig 2), corresponding to an increase in the prevalence of HMV from 7 to 33 per 100,000 inhabitants.

### Diagnosis Spectrum for the Primary Cause of HMV Therapy

**Figure 3:**
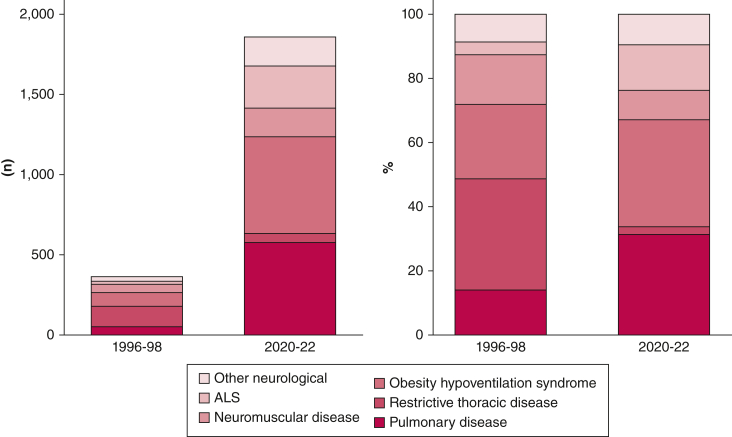
The main diagnosis groups in patients initiating home mechanical ventilation in Sweden over two periods (1996-1998 and 2020-2022), in absolute and relative numbers. ALS = amyotrophic lateral sclerosis.

In the early days of the registry, RTD was the predominant indication for HMV. However, parallel with decreasing prevalence, the proportion of HMV initiated for RTD decreased from 35% during 1996 to 1998 to 3% in 2020 to 2022 (Fig 3). In contrast, HMV initiated for lung disease, OHS, and ALS has increased, respectively, corresponding to 34%, 31%, and 14% of incident HMV in 2020 to 2022 (Figs 3A, 3B). Of note, 18.0% of those with lung disease had OHS as an additional diagnosis, and 22.0% of those with OHS had comorbid lung disease.

### Mode of Ventilation

Overall, during the study period, 4% of the patients were ventilated via a tracheostomy at the initiation of HMV (Table 3). The proportion of patients with invasive ventilation decreased from 9% to 2% between the first 5 and the last 5 years of the study period, but the number of patients with invasive ventilation did not change. No decrease in the use of invasive ventilation could be shown in patients with ALS. Only a fraction of patients with OHS started invasive ventilation therapy during the study period.

**Table 3:** Patients on Invasive Home Mechanical Ventilation as Part of the Entire Cohort (n = 381) and Subdivided for the Time Periods 1996 to 2000 (n = 56) and 2018 to 2022 (n = 56) Stratified by Diagnosis Groups

Time Period	Lung Disease	RTD	OHS	NMD	ALS	Other Neurology	Total
All patients in the cohort							
1996-2022	2,380	795	3,726	1,099	1,490	1,065	10,555
Patients on invasive ventilation, 1996-2022	25 (1.1)	27 (3.6)	19 (0.5)	90 (8.5)	69 (4.9)	151 (14.9)	381 (3.8)
Patients on Iinvasive ventilation, 1996-2000	4 (8.5)	11 (5.8)	NA	18 (19.8)	2 (6.5)	21 (42.9)	56 (9.4)
Patients on invasive ventilation, 2018-2022	2 (0.2)	NA	1 (0.10)	10 (3.1)	21 (4.9)	22 (7.8)	56 (1.8)
*P* value	< .001	.013	.719	< .001	.706	< .001	< .001

Data are presented as No. (%), No., or as otherwise indicated. ALS = amyotrophic lateral sclerosis; NA = not applicable; NMD = neuromuscular disease; OHS = obesity hypoventilation syndrome; RTD = restrictive thoracic disease.

### Patient Characteristics, Lung Function, and Blood Gases at Baseline

Table 1 presents the baseline characteristics at the start of HMV for 2020 to 2022. e-Table 1 shows the characteristics of all patients during the study period. e-Table 2 compares the characteristics for the first 3 years of the study (1996-1998) with those from 2020 to 2022. The overall proportion of women starting HMV increased slightly during the study period to 54% in 2020 to 2022. At the end of the study period, the proportion of women was highest in patients with lung disease (68%) and RTD (70%), and increased significantly during the study period in those with RTD from 54% to 71% (*P* = .040) and OHS from 35% to 53% (*P* = .003). No significant changes in sex distribution over time were shown in patients with lung disease, NMD, ALS, and other neurologic disorders.

**Figure 4:**
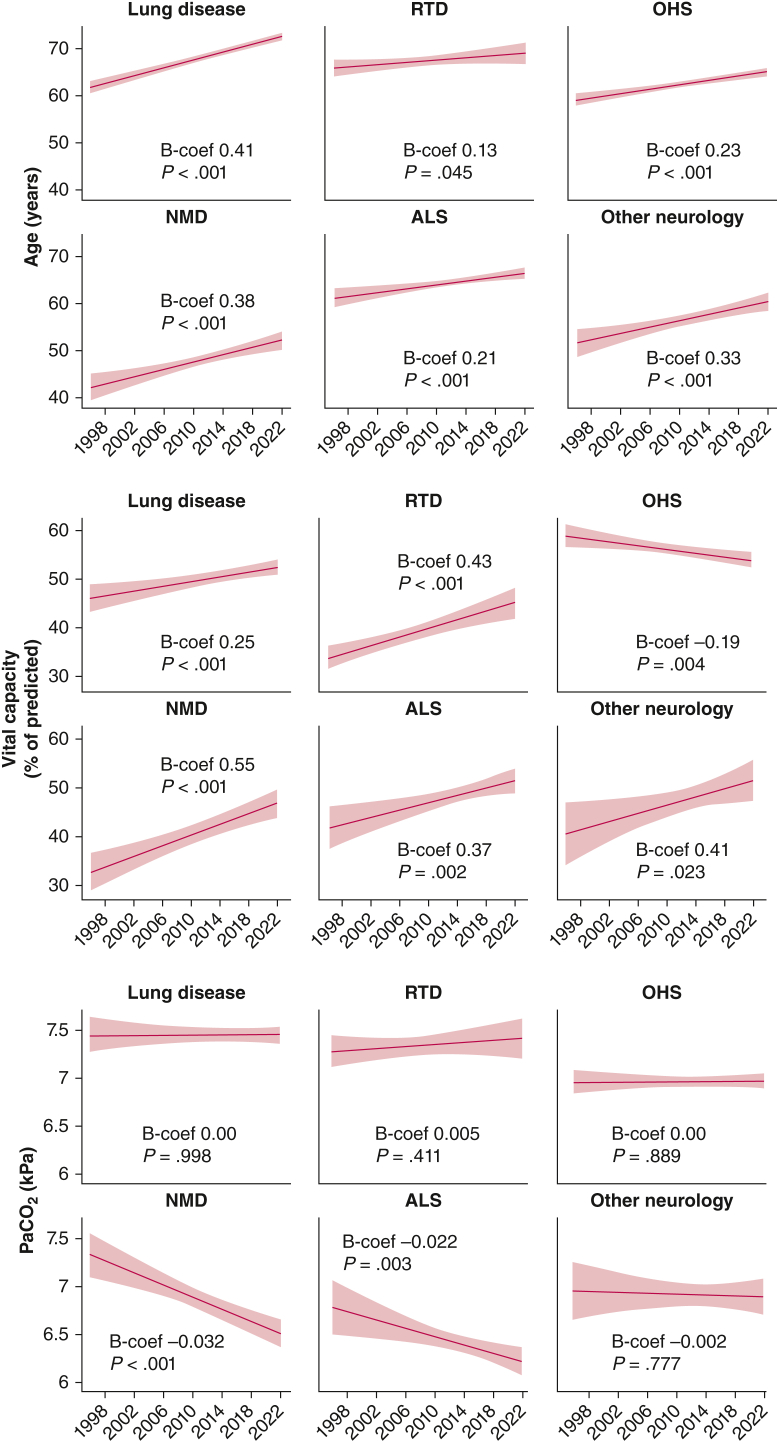
Time trends in age, vital capacity, and Paco_2_ at home mechanical ventilation initiation between 1996 and 2022 in Sweden. Linear regression analyses are stratified by diagnosis group. ALS = amyotrophic lateral sclerosis; β-coef = beta-coefficient; NMD = neuromuscular disease; OHS = obesity hypoventilation syndrome; RTD = restrictive thoracic disease.

Patients with lung disease and RTD were the oldest (Table 1), and during the study period, the overall age of patients initiating HMV increased from 58 [SD 15] years (1996-1998) to 65 [SD, 14] years (2020-2022) (*P* < .001). During the first 3 years of the registry, 0.8% of those starting HMV were ≥ 80 years of age; in 2020 to 2022, this proportion increased to 11.2% (*P* < .001). The changes in age at the start of HMV over the time period 1996 to 2022 are shown in Figure 4. The average age of patients initiated on HMV increased most in patients with lung disease, NMD, and other neurologic disorders over the study period (Fig 4).

BMI increased in those with OHS from 40 [SD 10] in 1996 to 1998 to 43 [SD 9] kg/m^2^ in 2020 to 2022 (*P* = .0048), and in those with NMD from 24 [SD 7] to 27 [SD 5] kg/m^2^ (*P* = .035). In the linear regression model, BMI decreased during the study time in patients with lung disease. No differences over time were shown for RTD, ALS, and those with other neurologic disorders (e-Fig 1).

VC at HMV initiation increased significantly in all diagnosis groups except for patients with OHS (Fig 4), where both VC and FEV_1_ declined over the study period. FEV_1_ at HMV initiation increased in those with RTD and NMD (e-Fig 1, Fig 4).

Of the patient groups, patients with ALS had blood gas values closest to normal at the initiation of HMV, whereas those with lung disease had the most deteriorated blood gases (Table 1). Initial Paco_2_ in those with NMD and ALS decreased during the study period to 6.6 [SD 1.2] and 6.3 [SD 1.3], respectively, in the last 3 years. No significant time trends for Pao_2_ or Paco_2_ were found in the other diagnosis groups (e-Fig 1, Fig 4).

## Discussion

The main findings of this large nationwide longitudinal cohort study include the following. First, patients on HMV today are almost 7 years older at initiation compared with 27 years ago. Second, the incidence of HMV therapy increased threefold during this period, and the prevalence of patients with HMV increased sixfold, illustrating the success of treatment and the increased demand for caregivers to provide long-term support to this patient group. Third, the leading indications of incident HMV have shifted from RTD to lung disease and obesity-related respiratory failure, reflecting the gain in evidence for HMV therapy for these groups. Fourth, HMV treatment is generally started earlier in the disease trajectory in patients with better lung function and, for some diagnosis groups, also better blood gas measurements at treatment start. Finally, the use of invasive ventilation is decreasing.

HMV incidence and prevalence are increasing in Sweden and internationally.^12^, ^13^, ^14^, ^15^^,^^20^^,^^21^ Several factors may have caused the overall increase of HMV treatment in Sweden during the study period. Obesity has increased in Sweden, which explains the higher number of patients with OHS treated with HMV.^5^ Scientific evidence related to the beneficial outcomes of HMV treatment in hypercapnic respiratory failure has created a growing awareness to identify and refer patients to pulmonary clinics for respiratory support treatment. In addition, randomized controlled trials have highlighted the benefit of HMV in patients with COPD with stable hypercapnic respiratory failure, leading to a new indication group.^9^^,^^10^ In contrast, financial incentives are less likely to boost the number of HMV treatments in Sweden because HMV treatment creates increased costs without dedicated budgets in publicly funded Swedish hospitals.

The Swedish incidence and prevalence numbers were 7 and 33 per 100,000 inhabitants, respectively. The prevalence rates vary between countries. In Lombardy in Italy, the prevalence of HMV has been estimated to be 63 per 100,000 people^22^; in the Geneva Lake area, 38 per 100,000 inhabitants^12^; in Finland, 35 per 100,000 inhabitants^23^; in New Zealand, 33 per 100,000 inhabitants^14^; in The Netherlands, 22 per 100,000 inhabitants^21^; and in Norway, 56 per 100,000 inhabitants.^24^ In a Canadian study, incident HMV increased from 2 to 5 per 100,000 inhabitants between 2000 and 2013.^20^ The rates are, however, difficult to compare because the cohorts are from different periods. Some studies include children,^13^^,^^24^, ^25^, ^26^, ^27^ some do not,^14^^,^^20^^,^^21^^,^^23^^,^^28^ and in others, it is not clear whether children are included or not.^12^^,^^15^^,^^22^^,^^29^ Swedevox has had an approximate coverage of 85%. Parallel with the COVID-19 pandemic, the number of reported patients on HMV in the registry has decreased, and there are also other signs of lower quality of reported data.^30^ Hence, the coverage can be somewhat lower now with underestimating incidence and prevalence numbers. Coverage estimations are only exceptionally reported in other cohorts.^24^

During the first years of the registry, RTD and NMDs were the dominating indications for HMV in Sweden. Diagnosis distribution in patients initiating HMV has changed over time, and OHS and lung disease are now the leading indications, corresponding to approximately one-third of each. According to longitudinal and cross-sectional studies, OHS and pulmonary diseases are also the top indications for HMV in Switzerland, Finland, Norway, and New Zealand.^12^^,^^14^^,^^23^^,^^24^ Interestingly, in The Netherlands, NMD and RTD are still the leading indications for HMV, whereas COPD and sleep-related breathing disorders only in the recent few years have begun to increase in prevalence.^21^ This explains the relatively low Dutch prevalence rate. In Poland, obstructive lung disease has become the leading indication for HMV.^13^ Between 2009 and 2019, the proportion of patients on HMV in Poland with NMD decreased from 45% to 34%, whereas OHS increased from a modest 1% to 5%. In a South Korean cohort from 2000 to 2009, NMDs, including ALS, corresponded to 87% of all patients on HMV, reflecting different treatment traditions.^25^ In Hungary, results from a web survey in 2018 reported an HMV prevalence of 3 per 100,000, of which 60% were diagnosed with central hypoventilation syndrome.^27^ A low response rate and possible selection bias limited this study.

In an international comparison, few patients on HMV in Sweden are ventilated invasively. This is probably due to treatment traditions and different patient profiles.^13^^,^^20^^,^^21^ As in other countries, the proportion of noninvasively treated patients is increasing with the development of better masks and cheaper yet more sophisticated ventilators for home use. Accordingly, we see a decrease in invasive ventilation in all diagnosis groups but ALS.

Our finding that the average age has increased at HMV starts aligns with a published study from Poland based on national fund-held data.^13^ In the current cohort, 12% of the patients in 2020 to 2022 were aged > 80 years. This proportion was even higher in a Swiss cohort from 2000 to 2015 at 18%. This may be because life expectancy has increased, and people are healthier up to older ages.^31^ Technical progression has also made treatment with HMV more effective, cheaper, and available to broader patient groups.

The general increase in VC at HMV initiation probably reflects an extension of the HMV indication. Physicians may be prone to prescribe HMV to a wider range of conditions, and not only the most severe cases become subject to treatment. A decrease in BMI in those with lung disease was observed, possibly due to the extension of the HMV indication.

Patients with OHS on HMV have increased weight and worsened lung function at HMV start. A possible partial explanation can be that less severe cases of OHS now, to an increased extent, are treated with CPAP instead of with HMV.^8^ Patients with NMD initiating HMV have increased in weight during the study period. A partial explanation could be that patients with Duchenne muscular dystrophy nowadays are recommended high doses of corticosteroids, with weight gain as an adverse effect.^32^

Paco_2_ at HMV initiation has decreased significantly. This is in accordance with new clinical guidelines advocating frequent clinical control in those with progressive neuromuscular conditions to find early signs of respiratory deterioration, especially in those with rapid progressive motor neuron diseases.^33^

### Proposed Future Research

The DISCOVERY cohort offers unique abilities to study the disease trajectory, including mortality and other outcomes, in patients with respiratory insufficiency. Future studies will focus on mortality in general in patients with HMV; on time trends, geographic disparities within Sweden, and mortality in patients with ALS with noninvasive and invasive ventilation; and on end-of-life care in patients with both ALS and with invasive ventilation in general in Sweden. Other studies will deal with health economic aspects of HMV, primarily in patients with ALS, but also in other diagnosis groups. We are also planning for a study with a more in-depth analysis of the disease trajectory, mortality, and time trends in patients on invasive ventilation with diagnoses other than ALS.

### Strengths and Limitations

The strength of this study is the uniquely large cohort size and the long study time, which spans over 27 years. The accuracy of the data in Swedevox is good. A published validation study showed high internal validity^34^; the coverage is estimated to be 85% to 90%.^17^ To our knowledge, this study is the first to systematically analyze time trends in patient characteristics, including anthropometrics and physiological data of patients initiating HMV. Published HMV cohort studies are more minor, up to 4,670 patients, often much less, and have shorter time spans, ranging from 4 to 15 years.^12^^,^^13^^,^^15^^,^^20^^,^^23^ In these studies, time trend data are scarce and restricted to diagnosis distribution^12^^,^^13^ and information on invasive ventilation.^13^^,^^20^ Some limitations must be considered. In the early days of the registry, the coverage probably was lower, with the risk of data not being fully representative. The categories of devices are only defined as devices for noninvasive or invasive ventilation. Information about ventilation modes, brands of ventilators, or changes in ventilator settings over time were unavailable in the registry. However, treatment followed national guidelines, which were rather stable during the past 2 decades.

## Interpretation

Our results show that in the 27 years to 2022, the incidence and prevalence of HMV in Sweden have increased markedly, patient demographics have changed, and use of invasive ventilation has decreased. The average age of patients initiated on HMV has increased, but treatment is started earlier in the disease trajectory.

## Funding/Support

A. P. was supported by the 10.13039/501100003793Swedish Heart-Lung Foundation [Grant 20230392] and ALF (the agreement concerning the research and education of doctors) [Grant ALF-979044]. L. G. was supported by the Swedish Heart and Lung Foundation [Grants 20180567, 20210529] and ALF [Grants GBG725601, GBG966283]. J. E. was supported by ALF funding [Grant 2022-Projekt 0016]. M. L. was supported by the Swedish Heart and Lung Foundation [Grant 20220686] and ALF [Grant ALF-979044]. J. S. was supported by ALF funding in Region Örebro County [Grant OLL-939092]. M. E. was supported by an unrestricted grant from the 10.13039/501100004359Swedish Research Council [Grant 2019-02081].

## Financial/Nonfinancial Disclosures

The authors have reported to *CHEST Pulmonary* the following: A. P. reports lecturing activities for ResMed, outside the topic of the current study. L. G. reports lecturing activities for Astra Zeneca, Lundbeck, and Resmed, outside the topic of the current study; is a medical advisor for the sleep diagnostic company, Onera; and is the coinventor of a patent related to sleep apnea treatment licensed to Desitin GMBH, Hamburg. J. S. has received personal fees from AstraZeneca, Boehringer Ingelheim, Chiesi, Takeda, and Novartis, unrelated to this work. M. E. has received a research grant from ResMed, and has received personal fees from AstraZeneca, Boehringer Ingelheim, Novartis, and Roche, unrelated to this work. None declared (J. E., D. H., M. L.).

## Figures and Tables

**e-Figure 1:**